# Case Report: Effectiveness of Inetetamab combined with immunochemotherapy as first-line treatment in two cases of advanced gastric cancer with HER2 expression: a retrospective analysis

**DOI:** 10.3389/fonc.2025.1647218

**Published:** 2025-10-27

**Authors:** Shan Wu, Miao Zhang, Huaqing Wang

**Affiliations:** ^1^ Department of Oncology, Tianjin Union Medical Center of Nankai University, Tianjin, China; ^2^ Tianjin Cancer Institute of Integrative Traditional Chinese and Western Medicine, Tianjin Union Medical Center, The First Affiliated Hospital of Nankai University, Tianjin, China; ^3^ The Institute of Translational Medicine, Tianjin Union Medical Center of Nankai University, Tianjin, China

**Keywords:** HER2-positive gastric cancer, Inetetamab, partial response (PR), safety and efficacy, immunochemotherapy

## Abstract

This retrospective study analyzed the effectiveness of Inetetamab combined with an immunochemotherapy regimen as first-line treatment in two cases of advanced gastric cancer with human epidermal growth factor receptor 2 (HER2) expression. Both patients were elderly males diagnosed with gastric cancer presenting with distant metastasis at initial diagnosis. They were treated with a combination of Inetetamab, Tislelizumab, and the XELOX regimen (Inetetamab 300mg administered on Day 1; Tislelizumab 200mg administered on Day 1; Oxaliplatin 150mg administered on Day 2; Capecitabine 1.5g orally twice daily on Days 1-14; repeated every 3 weeks per cycle). Efficacy evaluation revealed that both patients achieved a partial response (PR). They attained progression-free survival (PFS) durations of 10 to 12 months. Treatment was well-tolerated through-out, with no occurrence of grade 3–4 adverse events. This therapeutic regimen provided significant survival benefits for these patients with advanced, multiply metastatic, HER2-positive gastric cancer. The findings of this study suggest a novel first-line treatment strategy for advanced gastric cancer, potentially improving treatment efficacy and quality of life for HER2-positive gastric cancer patients. Nevertheless, further clinical trials are warranted to validate the efficacy and safety of this treatment approach.

## Introduction

Gastric cancer represents a significant global health threat with high mortality. In 2022, approximately 968,000 new cases and 660,000 deaths were reported worldwide, ranking fifth globally in both incidence and mortality (1). China accounted for 37.0% (358,000 cases) of global incidence and 39.5% (260,000 deaths) of mortality (1), with a five-year survival rate of just 33% for advanced disease (1, 2). Notably, nearly 50% of global gastric cancer-related deaths occur in China (3).

The human epidermal growth factor receptor 2 (HER2), a key member of the ERbB receptor family, plays a pivotal role in driving tumor cell proliferation and metastasis (4). HER2-positive gastric cancer—a distinct molecular subtype with a prevalence of 8.8% in China—carries an exceptionally poor prognosis, demonstrating a five-year survival rate below 20% (5).

Given this poor prognosis and deepened understanding of HER2 signaling, molecularly targeted agents against HER2 have emerged. The landmark ToGA trial established that trastuzumab plus chemotherapy extends median overall survival by 2.7 months in HER2-positive gastric cancer (6), establishing this regimen as first-line standard. However, survival benefits remain modest compared to HER2-positive breast cancer. Inetetamab, a novel anti-HER2 monoclonal antibody independently developed in China, demonstrates significantly enhanced antibody-dependent cellular cytotoxicity (ADCC) relative to trastuzumab.

This study reports two cases of patients with HER2-amplified metastatic gastric cancer who received first-line therapy with inetetamab combined with chemoimmunotherapy, achieving notable survival benefits exceeding 1 year. These cases provide novel clinical evidence for the targeted treatment of advanced HER2-positive gastric cancer and hold significant reference value for optimizing therapeutic strategies. The studies involving humans were approved by Tianjin Union Medical Center (Reference Number 2024-1).

## Case descriptions

### Patient 1

A 74-year-old male presented at Tianjin Union Medical Center of Nankai University

**Figure 1:**
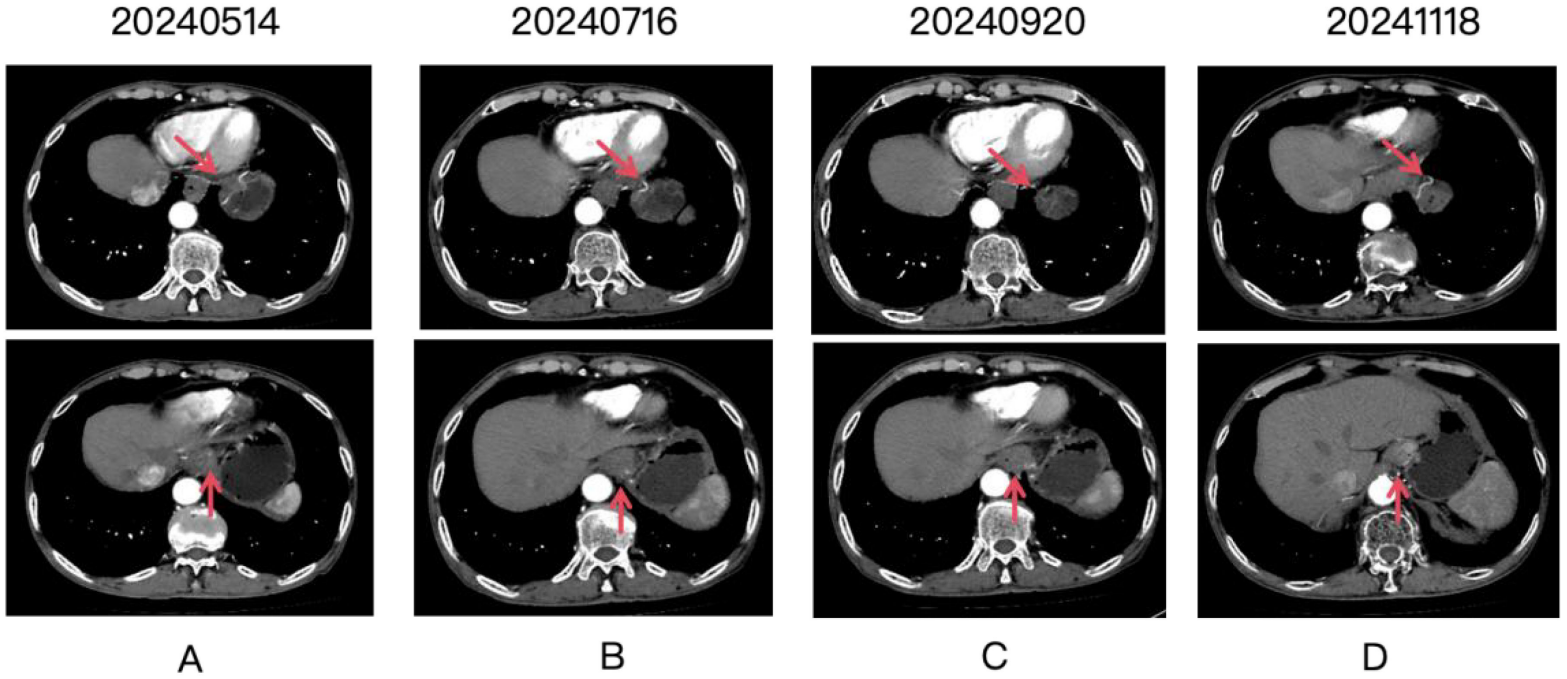
Four panels labeled **(A-D)** show CT scan images over time, with dates 20240514, 20240716, 20240920, and 20241118. Each panel contains two images. Red arrows point to the gastric lesion, which shows a partial response after treatment.

In February 2024, with progressive weight loss, fatigue, and anorexia. Endoscopic biopsy (February 28, 2024) revealed adenocarcinoma of the gastroesophageal junction/cardiac fundus. Subsequent pathological consultation at Tianjin Cancer Hospital (March 12, 2024; H2402290) confirmed poorly differentiated adenocarcinoma at this location. Immunohistochemistry (IHC) demonstrated: HER2 (1+); Mismatch repair proteins: MLH1 (+), MSH2 (+), MSH6 (+), PMS2 (+); Combined Positive Score (CPS) = 2. On March 13, 2024, the patient was evaluated at Tianjin Union Medical Center of Nankai University. PET-CT (Waterdrop Medical Diagnostics Center) revealed: Focal wall thickening at the gastroesophageal junction/gastric fundus and lesser curvature with intense radiopharmaceutical uptake, consistent with gastric carcinoma with perigastric fatty infiltration and lymph node involvement. A 1.2 × 1.0 cm nodule adjacent to the descending aorta in the right lower lung posterior basal segment showing intense radiopharmaceutical uptake, suggestive of metastatic disease. Molecular profiling (Putihe Biotech; March 18, 2024) identified: ERBB2 amplification (copy number: 1.98-fold) No detectable mutations in MLH1, MSH2, MSH6, or PMS2. Based on comprehensive imaging and pathological findings, the patient was diagnosed with stage IV gastric adenocarcinoma with pulmonary metastasis, with molecular confirmation of HER2 gene amplification. First-line therapy was initiated in May 2024 with the following regimen: Inetetamab (300 mg IV, day 0), Tislelizumab (200 mg IV, day 0), Oxaliplatin (150 mg IV, day 1), Capecitabine (1.5 g twice daily, days 1-14). The patient completed 6 cycles of this combination therapy, with follow-up imaging demonstrating a partial response (**Figure 1**). Assessment of treatment safety revealed an absence of severe (Grade 3-4) toxicities. The only treatment-emergent adverse events were mild (Grade 1) allergic reactions, including transient rash, which resolved without intervention. Subsequently, maintenance therapy with inotuzumab ozogamicin plus tislelizumab was administered for 4 additional cycles. As of the last follow-up in February 2025, the patient remained progression-free, achieving a progression-free survival (PFS) of 12 months.

### Patient 2

**Figure 2:**
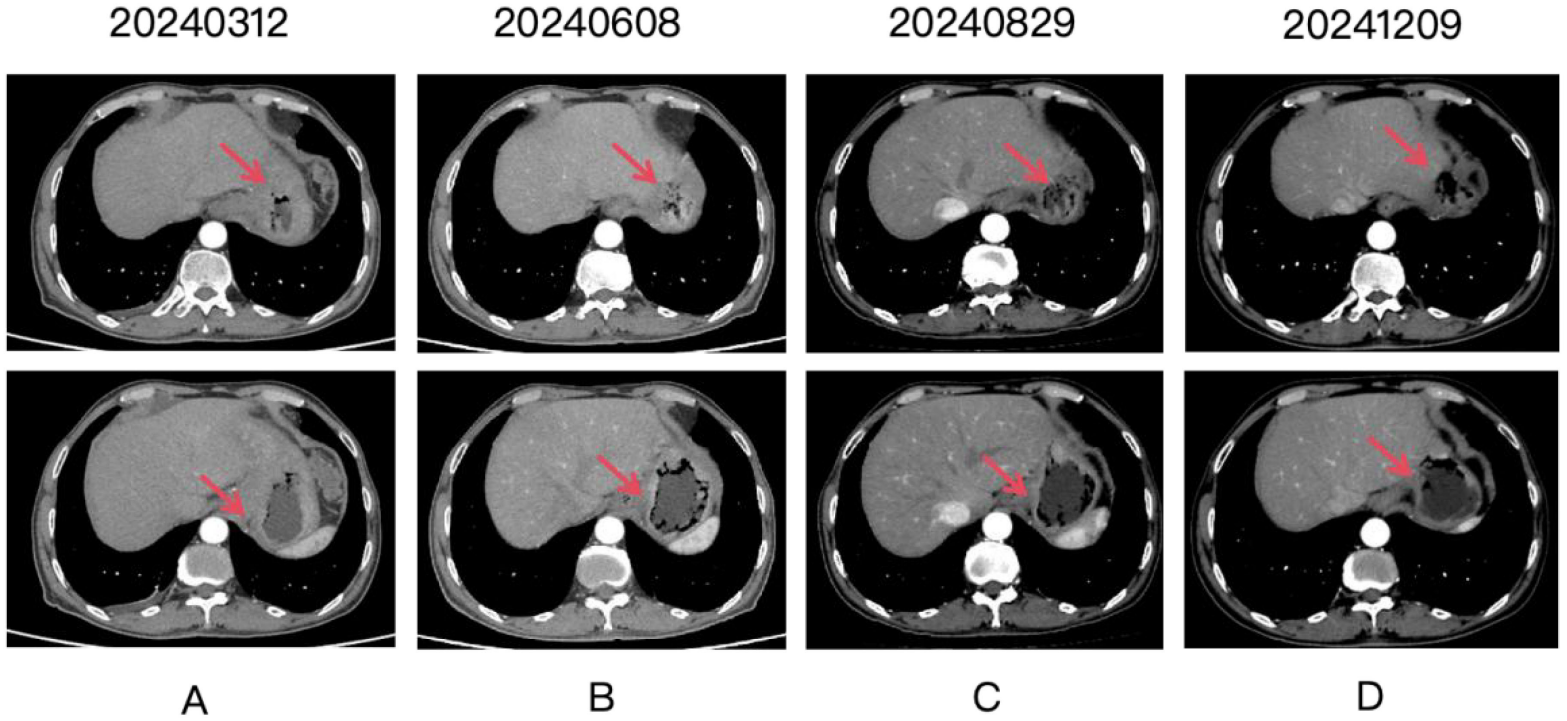
Four panels labeled **(A-D)** show CT scan images over time, with dates 20240312, 20240608, 20240829, and 20241209. Each panel contains two images. Red arrows point to the gastric lesion, which shows a partial response after treatment.

**Figure 3:**
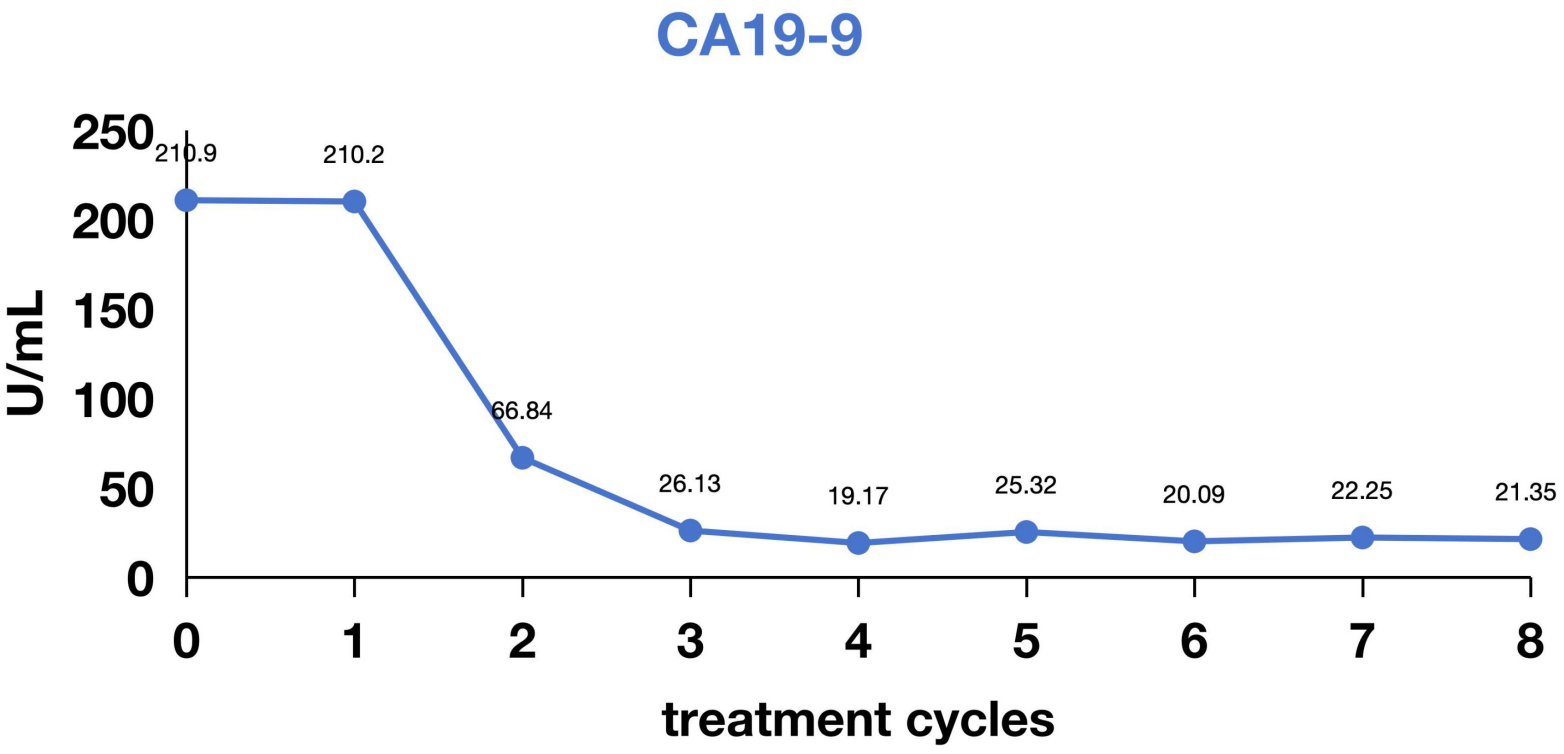
Variation in cancer antigen 199 levels during treatment.

**Figure 4:**
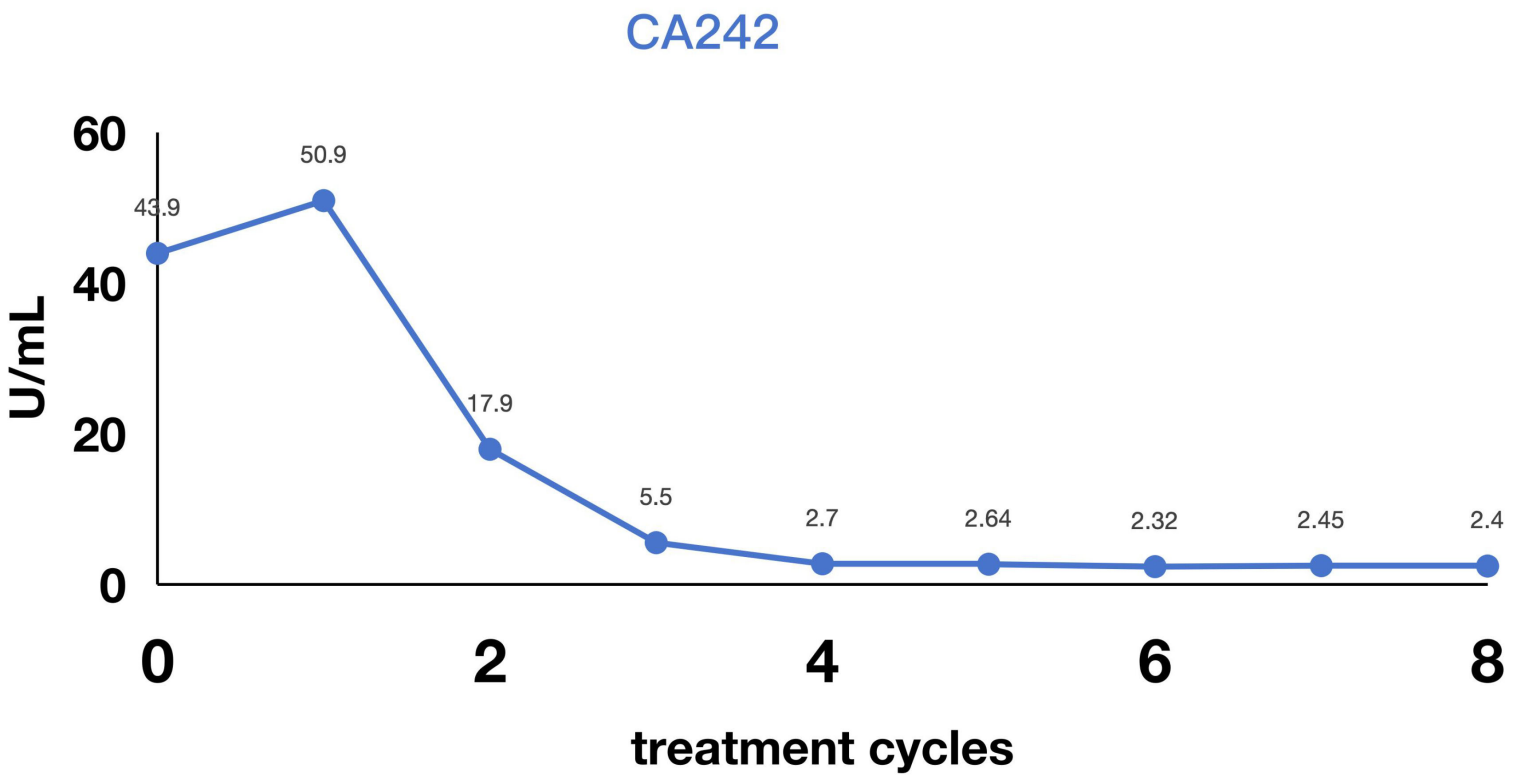
Variation in cancer antigen 242 levels during treatment.

A patient undergoing routine medical examination in March 2024 presented with upper gastrointestinal bleeding on gastroscopy (March 11, 2024), revealing: Mucosal congestion, edema, and ulceration at the gastroesophageal junction and gastric fundus/corpus (suggestive of linitis plastica), pending pathological confirmation. Pathological diagnosis of poorly differentiated adenocarcinoma (biopsy from greater curvature of gastric body). Immunohistochemistry (IHC): CK (Broad Spectrum+), CEA (+), p53 (focal weak positivity), Ki-67 (>80% positivity), CDX-2 (focal weak positivity). MMR proteins: MSH2(+), MSH6(+), MLH1(+), PMS2(+). HER-2 (heterogeneous expression: partial 2+, partial 3+). Abdominal CT was obtained in Tianjin Union Medical Center of Nankai University (March 12, 2024): Gastric mass consistent with gastric carcinoma (cT3NxM1); Omental metastases with adherence to adjacent bowel loops; Hepatic metastases (Segments II, IV, VII; largest nodule: 0.7 cm); Enlarged right cardiophrenic angle lymph node (recommended follow-up); Minimal ascites; Benign cystic lesion in pancreatic tail (recommended follow-up); Hepatic cyst (Segment IV); Bilateral renal cysts. The patient was diagnosed with stage IV gastric adenocarcinoma with hepatic and omental metastases. First-line therapy commenced in March 2024 with: Inetetamab (300 mg IV, day 0), Tislelizumab (200 mg IV, day 0), Oxaliplatin (100 mg/m² IV, day 1), Capecitabine (1.5 g twice daily, days 1-14). Following 8 cycles of combination therapy, radiographic assessment confirmed a partial response (**Figures 2**–**4**). The patient subsequently received 3 cycles of maintenance therapy with inetetamab and tislelizumab. As of the last follow-up in March 2025, the patient remained progression-free with a progression-free survival (PFS) exceeding 12 months.

## Discussion

In recent years, the advent of precision medicine has positioned HER2-targeted therapy as a focal point in managing HER2-positive gastric cancer. Accumulating evidence confirms HER2 overexpression across multiple solid tumors, including breast, lung, ovarian, prostate, and colorectal carcinomas (7). Approximately 20% of gastric cancer patients exhibit HER2 overexpression or gene amplification (7, 8), a biomarker strongly associated with adverse prognosis and elevated recurrence risk.

Therapeutic targeting of HER2 has thus gained increasing importance in gastric oncology, driving extensive research into novel anti-HER2 agents. Clinical trials of trastuzumab deruxtecan (T-DXd) demonstrate significant survival benefits in advanced gastric cancer, supporting its potential as a first-line option for HER2-positive disease. However, challenges remain regarding financial toxicity and the emergence of acquired resistance.

Inetetamab (Cipterbin^®^), China’s first domestically developed humanized anti-HER2 monoclonal antibody, has received regulatory approval for breast cancer. Its application in gastric cancer shows promising therapeutic potential. Current evidence indicates inetetamab exerts dual mechanisms of action: Inhibition of HER2 signaling pathways, Enhanced antibody-dependent cellular cytotoxicity (ADCC). This bifunctional activity represents a novel strategic approach in gastric cancer treatment.

Our manuscript evaluates the clinical efficacy and safety of a novel targeted therapy for gastric cancer, aligning with Frontiers in Oncology’s focus on translational and clinical advancements in gastrointestinal malignancies. By presenting data from a clinical course, this study directly addresses the journal’s emphasis on innovative therapeutic strategies, precision oncology, and improved patient outcomes in gastric cancer, a field of critical unmet need. Our findings contribute to the evolving landscape of targeted treatments, offering potential relevance to researchers and clinicians in the Gastric Cancers specialty section.

### Efficacy and safety validation in first-line therapy

Clinical data presented at the 2024 ESMO ASIA Congress and ASCO Annual Meeting demonstrate that inetetamab combined with the SOX regimen (S-1 plus oxaliplatin) achieves an objective response rate (ORR) comparable to trastuzumab (the conventional anti-HER2 agent) in first-line treatment of HER2-positive advanced gastric cancer, with a median progression-free survival (mPFS) of 8.5 months and favorable tolerability (9–11). These findings suggest that structural optimizations (e.g., Fc segment modification) in inetetamab may preserve the therapeutic efficacy of traditional anti-HER2 agents while mitigating cardiac toxicity and infusion reaction risks. Notably, Asian subgroup analyses revealed a significantly higher ORR of 50% (9), potentially attributable to molecular subtypes of HER2-positive gastric cancer (e.g., higher prevalence of intestinal-type per Lauren classification) and pharmacogenetic variations. However, there are a couple of research limitations in the therapy as well: 1.Methodological Constraints: Most evidence derives from single-arm Phase II trials lacking randomized controlled designs versus trastuzumab, leaving differences in survival benefits (e.g., overall survival [OS]) unresolved. 2.Statistical Power Deficits: Limited sample sizes (typically <100 patients) preclude robust subgroup analyses (e.g., by PD-L1 expression status or HER2 amplification levels). Nevertheless, these preliminary results establish a critical foundation for Phase III clinical trial development.

### Exploratory advances in second-line and beyond therapy

Early-phase investigations and case reports have revealed the therapeutic potential of inetetamab in later-line settings. Notably, a documented case of HER2-positive advanced gastric cancer achieved a progression-free survival (PFS) of 17 months with second-line inetetamab plus chemotherapy—substantially exceeding the historical median PFS of 3–4 months for conventional second-line regimens (9, 12). This exceptional response may stem from ADCC-enhanced tumor microenvironment remodeling: Inetetamab engages CD16 receptors on natural killer (NK) cells through its optimized Fc domain, activating immune-mediated tumor lysis and thereby counteracting chemotherapy-induced immune evasion (12). While *in vitro* studies strongly suggest that the efficacy of Inetetamab is driven by enhanced ADCC, our current clinical data lack direct immunohistochemical evidence of NK cell infiltration in the tumor microenvironment of the presented patients. In the context of our patients, the strong clinical response observed in both patients could be hypothetically explained by a robust pre-existing NK cell infiltrate. Future studies with paired tissue sampling are warranted to confirm this correlation for Inetetamab. To truly validate the role of ADCC *in vivo*, prospective studies monitoring dynamic changes in NK cell activity and tumor immune microenvironment composition during Inetetamab treatment are needed. However, there are methodological considerations and research gaps. 1. Generalizability Concerns: Such exceptional responses may reflect selection bias (e.g., HER2 hyperexpression or co-occurring genomic alterations) and require validation in larger cohorts. 2. Combination Strategy Uncertainty: Optimal therapeutic sequencing—particularly regarding integration with immune checkpoint inhibitors (ICIs)—remains undefined. Future studies should establish biomarker-guided combination frameworks.

The distinct therapeutic profile of inetetamab stems from deliberate molecular optimization. While its Fab fragment shares identical HER2-binding characteristics with trastuzumab (214 identical amino acids), strategic modifications to the Fc domain through amino acid sequence engineering and manufacturing process refinements yield critical enhancements. Specifically: Fab segment: Maintains high-affinity HER2 binding Fc segment: Exhibits enhanced antibody-dependent cellular cytotoxicity (ADCC) via reduced core fucosylation (≈40% decrease) (13). Quantitative analyses confirm an 11.1% increase in ADCC potency versus trastuzumab (14), a feature particularly consequential in gastric cancer’s immunosuppressive microenvironment—characterized by tumor-associated macrophage (TAM) infiltration and abundant immunosuppressive cytokines (e.g., IL-10, TGF-β). Enhanced ADCC activity may counteract this immunosuppression by augmenting innate immune activation. Recent preclinical studies elucidate additional pathways: 1.Pyroptosis Induction: In HER2-positive models, inetetamab plus cisplatin activates the Gasdermin E pathway, triggering pyroptosis with concomitant release of pro-inflammatory cytokines (e.g., IL-1β, IL-18) that promote immune cell infiltration (15). 2.Immune Microenvironment Reprogramming: Treatment significantly elevates CD8+ T cell density within tumor-infiltrating lymphocytes (TILs) while reducing immunosuppressive populations (Tregs: ↓28%; MDSCs: ↓34%), indicating intrinsic immune-potentiating effects (15). These mechanistic insights provide a rationale for combinatorial therapeutic strategies.

Despite promising therapeutic potential, the clinical implementation of inetetamab faces significant hurdles: 1. Unapproved Indication Constraints: Current regulatory approval restricts inetetamab to breast cancer, rendering its use in gastric cancer off-label. This poses ethical-legal dilemmas for clinicians and imposes substantial financial toxicity on patients due to reimbursement limitations. Although the 2024 ESMO ASIA data demonstrated non-inferiority to trastuzumab, regulatory agencies typically require Phase III survival endpoints (e.g., overall survival [OS]) for formal approval—a threshold not yet met by existing studies. 2. Predictive Biomarker Deficits: High heterogeneity within HER2-positive gastric cancer results in marked response variability. Current protocols rely solely on HER2 IHC 3+ or FISH positivity for patient selection, yet primary/secondary resistance persists in ≈30% of cases. In our study, patient 1 showed HER2 IHC 1+ but ERBB2 amplification (Gene Amplified). This is a classic example of genetic heterogeneity. The gene amplification test confirms that the HER2 gene is amplified in the cells analyzed. However, the protein translation or post-translational processing is inefficient, resulting in only low-level (1+) protein expression on the cell surface. Therefore, selecting Inetetamab is a valid and targeted choice. The response is theoretically possible but likely suboptimal. The number of HER2 receptors on the cell surface (low due to 1+ IHC) may be insufficient for robust antibody binding and effective ADCC. The patient might show a response, but the risk of early resistance due to the outgrowth of the low-expressing clones is high. This case highlights why ERBB2 gene expression is an important predictor for antibody-based therapies. Putative resistance mechanisms—including shedding of HER2 extracellular domain (ECD), PIK3CA mutations, and PTEN loss—lack validation as consensus biomarkers. Future frameworks must integrate multi-omics profiling (genomic/transcriptomic/proteomic) to develop predictive algorithms. 3. Unresolved Long-Term Safety: Phase II data indicate lower cardiotoxicity incidence versus trastuzumab (6% vs. 11%; p=0.04) (10), but longitudinal surveillance data (e.g., left ventricular ejection fraction [LVEF] trajectories) remain absent. Furthermore, enhanced ADCC activity may elevate risks of immune-related adverse events (irAEs) such as thyroid dysfunction (incidence: 8-12%) or colitis, necessitating vigilant monitoring in Phase III trials.

Our observations are from a small, specific demographic (elderly men) and that age-related factors were not a primary focus of this analysis. A compromised immune system may theoretically diminish the effectiveness of antibody-dependent cellular cytotoxicity (ADCC), a key mechanism of action for anti-HER2 mAbs, which relies on functional effector cells like natural killer (NK) cells (16). However, the preserved efficacy in our cases suggests that this mechanism may remain operative in some older patients. The positive initial experience with Inetetamab in these elderly men is encouraging and suggests it may be a viable therapeutic option for a broader, older patient population. However, the inherent fragility of this demographic necessitates caution. Rather than basing decisions on age alone, a comprehensive geriatric assessment is essential to accurately gauge individual resilience and risk. Future prospective studies and subgroup analyses of larger datasets should specifically focus on the efficacy and safety of Inetetamab in older adults, stratifying patients by GA domains to truly understand its role in the geriatric oncology landscape.

Building upon current evidence and addressing existing challenges, the following strategic foci emerge:

1. Advancement of Phase III Clinical Trials: Implement multicenter randomized controlled trials (e.g., inetetamab + SOX vs. trastuzumab + SOX) with: Primary endpoint: Overall survival (OS); Secondary assessments: Quality of life (QoL) metrics and cost-effectiveness analyses; Prespecified subgroup analyses: PD-L1 expression, HER2 copy number variation, and tumor mutational burden (TMB) to identify beneficiary populations.
2. Optimization of Combination Strategies: Explore novel therapeutic synergies through dual-targeting approaches, small-molecule TKIs (e.g., pyrotinib), HER2/EGFR bispecific antibodies (Targeting bypass resistance pathways), Immunotherapy augmentation through, enhancing systemic anti-tumor immunity beyond PD-1 blockade, CTLA-4 inhibitors and oncolytic viruses.
3. Expansion into Perioperative Settings: Leverage insights from breast cancer neoadjuvant therapy (pathological complete response [pCR] rate: 66.7% with inetetamab-chemotherapy combinations) (17) to evaluate: Key surgical metrics, pathological response rates and R0 resection rates; long-term outcomes: Disease-free survival (DFS) and overall survival (OS).
4. Real-World Evidence Generation: Establish a national gastric cancer registry database to: document outcomes of off-label inetetamab use, provide pragmatic evidence for regulatory indication expansion.

This study reports two patients with unresectable, HER2-positive (IHC-confirmed or ERBB2-amplified) advanced gastric cancer who received first-line therapy with the domestically developed anti-HER2 monoclonal antibody inetetamab combined with PD-1 inhibition and XELOX chemotherapy. Following 8 cycles of chemoimmunotherapy, both patients maintained partial responses (PR). Subsequent maintenance therapy with inetetamab yielded progression-free survival (PFS) exceeding 12 months—surpassing historical benchmarks for this population. The regimen demonstrated favorable tolerability with no significant deterioration in quality of life (QoL) relative to pre-diagnosis baselines. High treatment adherence and sustained disease control were observed throughout. Moving forward, correlating clinical efficacy with patient-reported outcomes will be essential to fully define the therapeutic value of Inetetamab and ensure it not only improves survival but also maintains or enhances the quality of life for patients with HER2-positive metastatic breast cancer.

These findings indicate that inetetamab-based chemoimmunotherapy confers substantial clinical benefit in HER2-expressing gastric cancer. The enhanced antibody-dependent cellular cytotoxicity (ADCC) profile of this structurally optimized monoclonal antibody suggests broad therapeutic potential for advanced HER2-positive disease, warranting validation in larger clinical trials to establish evidence-based treatment paradigms. For HER2-positive metastatic gastric cancer, inetetamab-chemotherapy combinations demonstrate significant survival advantages and favorable safety profiles. As precision oncology advances, molecular characterization and tumor immune microenvironment analysis have emerged as critical research foci. In this context, targeted-immunotherapy combinations represent a promising frontier, exemplified by the KEYNOTE-811 trial: Pembrolizumab combination group: ORR 72.6% (overall); 73.2% (PD-L1 CPS≥1 subgroup). Placebo combination group: ORR 60.1% (overall); 58.4% (PD-L1 CPS≥1 subgroup) (18). Future clinical and translational studies will optimize these combinatorial strategies, refining therapeutic algorithms to improve survival outcomes and quality of life.

## Data Availability

The original contributions presented in the study are included in the article/supplementary material. Further inquiries can be directed to the corresponding author.
